# Sex-Specific Metabolic Effects of Dietary Folate Withdrawal in Wild-Type and *Aldh1l1* Knockout Mice

**DOI:** 10.3390/metabo12050454

**Published:** 2022-05-18

**Authors:** Jaspreet Sharma, Blake R. Rushing, Madeline S. Hall, Kristi L. Helke, Susan L. McRitchie, Natalia I. Krupenko, Susan J. Sumner, Sergey A. Krupenko

**Affiliations:** 1 Nutrition Research Institute, UNC Chapel Hill, Kannapolis, NC 28081, USA; sharmaj@email.unc.edu (J.S.); blake_rushing@unc.edu (B.R.R.); madeline_hall@unc.edu (M.S.H.); susan_mcritchie@unc.edu (S.L.M.); natalia_krupenko@unc.edu (N.I.K.); susan_sumner@unc.edu (S.J.S.); 2 Department of Nutrition, UNC Chapel Hill, Chapel Hill, NC 27599, USA; 3 Department of Comparative Medicine, Medical University of South Carolina, Charleston, SC 29425, USA; helke@musc.edu

**Keywords:** *Aldh1l1* knockout, folate metabolism, untargeted metabolomics, dietary folate restriction, liver, plasma

## Abstract

ALDH1L1 (10-formyltetrahydrofolate dehydrogenase), an enzyme of folate metabolism, is highly expressed in the liver. It regulates the overall flux of folate-bound one-carbon groups by converting 10-formyltetrahydrofolate to tetrahydrofolate and CO_2_ in a NADP^+^-dependent reaction. Our previous study revealed that *Aldh1l1* knockout (KO) mice have an altered liver metabotype with metabolic symptoms of folate deficiency when fed a standard chow diet containing 2 ppm folic acid. Here we performed untargeted metabolomic analysis of liver and plasma of KO and wild-type (WT) male and female mice fed for 16 weeks either standard or folate-deficient diet. OPLS-DA, a supervised multivariate technique that was applied to 6595 and 10,678 features for the liver and plasma datasets, respectively, indicated that genotype and diet, alone or in combination, gave distinct metabolic profiles in both types of biospecimens. A more detailed analysis of affected metabolic pathways based on most confidently identified metabolites in the liver and plasma (OL1 and OL2a ontology level) indicated that the dietary folate restriction itself does not fully recapitulate the metabolic effect of the KO. Of note, dietary folate withdrawal enhanced the metabolic perturbations linked to the ALDH1L1 loss only for a subset of metabolites. Importantly, both the ALDH1L1 loss and dietary folate deficiency produced sex-specific metabolic effects.

## 1. Introduction

**Figure 1:**
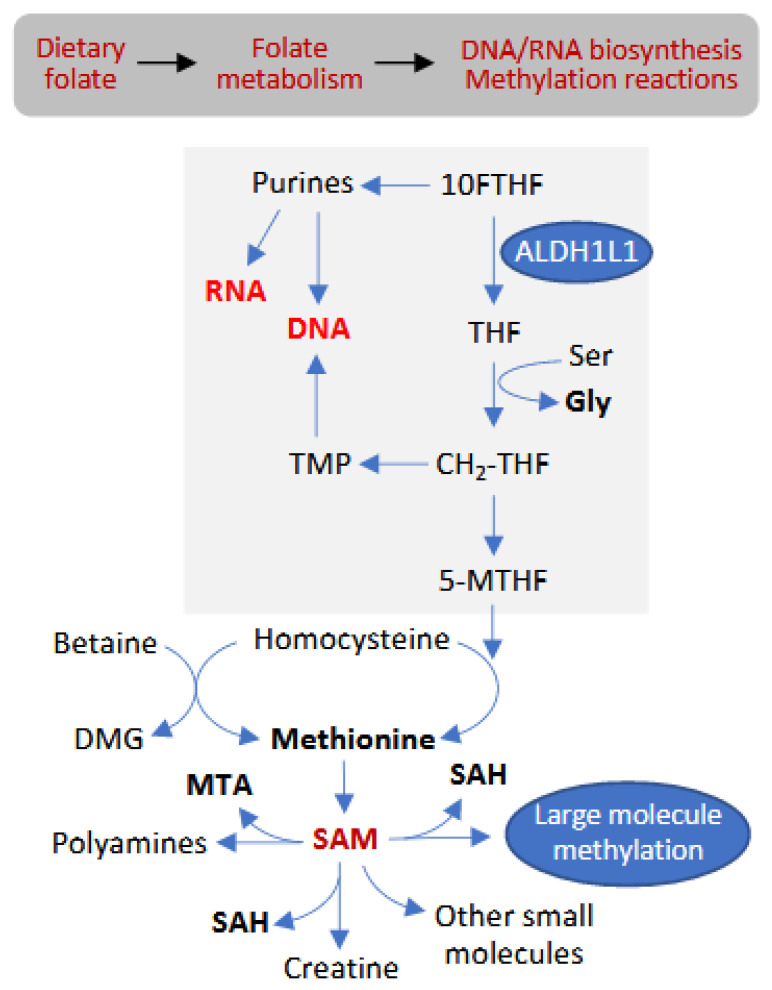
Disposition of ALDH1L1 in folate metabolism. ALDH1L1 reaction replenishes the THF pool, thus supporting glycine synthesis from serine; regulates de novo purine biosynthesis; and controls the overall flux of one-carbon groups through the folate pool.

Folate metabolism (simplistically depicted in Figure 1) maintains key cellular processes such as nucleotide and amino acid biosynthesis, with folate-dependent re-methylation of homocysteine to methionine being linked to the production of the universal methyl donor S-adenosylmethionine (SAM) [1,2]. Accordingly, efficient folate metabolism enables DNA/protein biosynthesis, as well as the variety of methylation reactions [3]. Humans cannot synthesize folate and, instead, obtain it from the diet, with dietary folate intake significantly influencing folate-dependent cellular processes. Insufficient folate intake ultimately leads to the deregulation of homeostasis and underlies certain diseases, most notably neural tube defects [4,5,6,7]. In 1996, the FDA approved a mandatory fortification of several types of grain foods in the US with a synthetic form of the vitamin, folic acid (FA), to prevent these birth defects. Besides dietary folate intake, however, folate metabolism is regulated by numerous enzymes involved in folate pathways [4]. 

One of the folate-metabolizing enzymes, ALDH1L1 (10-formyltetrahydrofolate dehydrogenase), converts 10-formyl-THF to THF (tetrahydrofolate) with simultaneous production of NADPH from NADP^+^ [8]. This enzyme is very abundant in the liver, comprising about 1% of total cytosolic protein in hepatocytes [8]. Several studies have indicated that ALDH1L1 functions as a key regulator of folate metabolism, clearing one-carbon groups (in the form of CO_2_) from the cell, thus controlling their flux to biosynthetic processes and methylation reactions (Figure 1) (reviewed in References [8,9]). Therefore, alterations of the expression of this enzyme can have a profound effect on cellular metabolism. The most striking example is the methylation-driven silencing of the *ALDH1L1* gene in human cancers [10,11,12,13]. The loss of ALDH1L1 protein positively correlates with the occurrence of malignant tumors, hence the suggestion that the enzyme is a putative tumor suppressor [10,11,12,13,14,15,16,17,18]. In line with this hypothesis, our recent study indicated that the loss of *Aldh1l1* in mice promotes more rapid growth of large tumors in the chemical carcinogenesis model of hepatocellular carcinoma [19]. Mechanisms underlying this effect were linked to several metabolic pathways which are beneficial for rapid proliferation [19]. 

In this regard, *Aldh1l1* knockout mice, both males and females, showed altered metabotypes with significant changes in numerous metabolites, most noticeably decreased levels of glycine and glycine conjugates [20]. These changes were associated with a decreased capacity of the knockout animals to generate sufficient levels of THF, which is required for the reaction producing glycine from serine. At certain physiological conditions, this reaction can be the source of up to 80% of glycine in humans [21]. Unexpectedly, *Aldh1l1* knockout (KO) mice displayed functional folate deficiency despite the fact that they were fed a folate-proficient diet, and the total levels of folate coenzymes were not changed in these mice [20]. The origin of such folate deficiency is the same as the decrease in glycine: insufficient levels of THF for several folate pathways requiring this coenzyme. 

The present study aimed to address two questions: (i) whether dietary folate restriction leads to the metabotype observed in *Aldh1l1* KO and (ii) whether dietary folate restriction exacerbates the metabotype associated with the loss of *Aldh1l1*. Toward these goals, we performed an untargeted metabolomic analysis of liver and plasma samples from wild-type (WT) and *Aldh1l1* KO mice, both male and female, fed a folate-adequate or folate-deficient diet and linked corresponding metabotypes to sex-specific histological and hematological features. We used both male and female mice because the literature indicates that sex is one of the most relevant biological variables [22,23,24,25], with sex-specific metabolic responses being reported in both gene knockout and dietary studies [26,27,28]. 

## 2. Materials and Methods

### 2.1. Animal Experiments

All animal experiments were conducted in strict compliance with the National Institutes of Health’s “Guide for Care and Use of Laboratory Animals” and were approved by the Institutional Animal Care and Use Committee at the David H. Murdock Research Institute (DHMRI), Kannapolis, North Carolina (protocol #20-005). Mice were housed in microisolator cages on a 12 h light/dark cycle and allowed access to water and chow ad libitum.

### 2.2. Generation of Aldh1l1 Knockout Mice

*Aldh1l1^−/−^* mice were generated as we previously reported [20] and were back-crossed (8–10 generations) to the C57Bl/6NHsd mice purchased from Envigo (Indianapolis, IN, USA). Heterozygous males and females were intercrossed to obtain knockout and wild-type littermates. 

### 2.3. Genotyping 

Genotyping was carried out by polymerase chain reaction (PCR) of tail lysates obtained by using direct PCR (Tail) lysis reagent (cat #101-T) and Proteinase K (Specific activity >600 U/mL, Thermo Scientific, Waltham, MA, USA, cat #EO0491). Primers for genotyping are shown in Supplementary File S1 (Table S1). Amplification generates 199 bp amplicon for the wild-type allele and 685 bp amplicon for the mutant allele. 

### 2.4. Administration of Diets

At 10–12 weeks of age, wild-type (WT, *Aldh1l1^+/+^)* and knockout (KO, *Aldh1l1^−/−^*) littermates, both male and female, were randomized to four groups: (1) *Aldh1l1^+/+^* on control diet, WT (CD); (2) *Aldh1l1^−/−^* on control diet, KO (CD); (3) *Aldh1l1^+/+^* on folate-deficient diet, WT (FDD); and (4) *Aldh1l1^−/−^* on folate-deficient diet, KO (FDD). The mice were fed synthetic isocaloric diets purchased from Envigo and containing 14.4% kcal from fat, 66.5% kcal from carbohydrates, and 19.1% kcal from protein. The two diets used in this study differ in the amount of folic acid (FA): FA-deficient diet (FDD, TD.95427) which contains only residual FA (0.2 ppm) from added proteins, and control diet (CD) with additional 2 ppm FA added (TD.04194). *Aldh1l1^+/+^* and *Aldh1l1^−/−^* littermates, both male and female, were placed on respective diets for 16 weeks. Body weight was recorded weekly until the end of the study. At the end of dietary exposure, mice were fasted for 4 h before being euthanized via CO_2_ asphyxiation and cervical dislocation. Whole livers were washed with ice-cold PBS and weighed. A consistent region of the left lateral lobe (150–250 mg) was taken for metabolomics analysis and flash-frozen in liquid nitrogen for further analysis. Blood was collected into EDTA-containing tubes via cardiac puncture. Plasma was separated by centrifugation at 2000× *g* for 10 min at 4 °C.

### 2.5. Histology

Individual organs from animals in all experimental groups were collected after euthanasia and weighed. Organ sections fixed in 10% formalin were embedded in paraffin tissue blocks, cut in 5 μm sections, and placed on slides. Tissue-section slides were deparaffinized, rehydrated and stained with Hematoxylin and Eosin according to standard protocols. Images were captured at 10× and 20× magnification.

### 2.6. Blood Analysis

Whole-blood aliquots were subjected to blood analysis, using an automated hematology blood counter (Sysmex pocH-100*i* Automated Hematology Analyzer). The parameters included glucose (mg/dL), hemoglobin (Hb, g/dL), erythrocytes (1 × 10^6^/μL), leucocytes (1 × 10^3^/μL), and platelet count (1 × 10^3^/μL), which were performed to rule out any hematological abnormalities developed due to low folate consumption.

### 2.7. Plasma Sample Preparation

Aliquots of 50 μL of plasma from each mouse were placed into pre-labeled 2.0 mL Lo-Bind Eppendorf tubes. Method blanks were prepared by aliquoting 50 μL of LC–MS grade water into 4 pre-labeled 2.0 mL Lo-Bind Eppendorf tubes and were processed identically to study samples. A solution of 80% methanol (containing 500 ng/mL Tryptophan-d5) was added at a volume of 400 μL to each tube. The volume of 80% methanol was adjusted if vials contained less than 50 μL of plasma to maintain the same ratio of plasma to extraction volume. Samples were mixed on a multi-tube vortexer for 2 min at 5000 rpm. Samples were incubated at 4 °C for 10 min, and protein was pelleted by centrifuging samples at 4 °C and 16,000× *g* for 10 min. A volume of 350 μL of supernatant was transferred to new tubes, and the tubes of supernatant were dried by SpeedVac overnight. Samples were reconstituted, using 100 μL of reconstitution solution (95:5 H_2_O: methanol). Samples were vortexed at 5000 rpm for 10 min on a multi-tube vortexer and centrifuged at 4 °C and 16,000× *g* for 10 min. Supernatants were transferred to HPLC autosampler vials and a total quality-control study pool (QCSP) was prepared from the final extracts by combining 5 μL of each study sample. Samples were analyzed by injecting 5 μL onto the LC–MS column for an untargeted analysis. 

### 2.8. Liver Sample Preparation

Liver extracts were prepared based on previous methods [29]. Liver samples were pre-weighed (120–150 mg) and placed into MagNA Lyser tubes containing ~50 beads. Cold homogenization solution, 5× (5 μL/mg of tissue) (80:20 methanol: H_2_O), was added to each tube, while keeping the samples frozen. Method blanks were prepared by adding 500 μL of homogenization solution to four empty MagNA Lyser tubes with beads and were processed identically to study samples. Samples were homogenized on an Omni Bead Ruptor Elite at 5 m/s for 30 s. Protein and tissue debris were pelleted by centrifuging samples at 4 °C and 16,000× *g* for 10 min. A volume of 200 μL of supernatant was transferred to new pre-labeled 2.0 mL low-bind Eppendorf tubes and dried by SpeedVac overnight. All samples were reconstituted by adding 500 μL of Tissue Reconstitution Solution (95:5 H_2_O:methanol with 500 ng/mL Tryptophan-d5) and vortexing at 5000 rpm for 10 min on a multi-tube vortexer, followed by centrifugation at 4 °C and 16,000× *g* for 10 min. Supernatants were transferred to autosampler vials, and 5 μL of each study sample was combined to make a total QCSP. An injection volume of 5 μL was used for untargeted LC-MS analysis.

### 2.9. UHPLC–High-Resolution Mass Spectrometry (UHPLC–HR-MS) Data Acquisition and Data Preprocessing

Metabolomics data were acquired with a Vanquish UHPLC system coupled to a Q Exactive™ HF-X Hybrid Quadrupole-Orbitrap Mass Spectrometer (Thermo Fisher Scientific, San Jose, CA, USA), using methods previously described [30,31]. The study samples (plasma or liver extracts) were randomized, and the blanks and QCSPs were inserted after every 10 study samples. Metabolites were separated via an HSS T3 C18 column (2.1 × 100 mm, 1.7 µm, Waters Corporation, Milford, MA, USA) at 50 °C, with binary mobile phase of water (A) and methanol (B), each containing 0.1% formic acid (*v/v*). The UHPLC linear gradient started at 2% B, was increased to 100% B in 16 min, and then was held for 4 min, with the flow rate at 400 µL/min. The untargeted data were acquired from 70 to 1050 *m*/*z*, using the data-dependent acquisition mode. Progenesis QI (version 2.1, Waters Corporation) was used for peak picking, alignment, and normalization. Background signals were filtered out by only analyzing peaks with a mean peak abundance fold change > 3 in the total QCSP vs. the blanks. Peaks were then normalized in Progenesis QI, using the “normalize to all” feature. This normalization strategy uses a QCSP to calculate a global scaling factor between samples which is used to normalize peak abundances [32]. For plasma data, signals that had an RSD > 30% across the total QCSPs were also removed. Two liver samples did not meet quality control standards, due to poor chromatogram shape/alignment, and were thus excluded from the analysis. 

### 2.10. Multivariate and Univariate Statistical Analysis of Untargeted UHPLC–HR-MS Data

Principal component analysis (PCA) and orthogonal partial least square discriminant analysis (OPLS-DA) were performed by using SIMCA 15 (Umetrics, Umeå, Sweden) on the normalized, preprocessed untargeted data for both plasma and liver samples. PCA visualizations were used to demonstrate clustering of the total QCSPs and blanks. OPLS-DA was used to determine differentiating peaks between each two-way comparison by calculating variable importance in projection (VIP) scores for each peak. Statistical analyses were conducted by using SAS 9.4 (SAS Institute Inc., Cary, NC, USA). The *p*-values were calculated for each peak, using the Exact Wilcoxon Rank Sum Test to identify significantly different peaks between each two-way comparison. Fold changes were calculated by using median values for peak abundances. Because this is an exploratory study with a modest sample size, *p*-values were calculated between pairwise comparisons and were not adjusted for multiple comparisons. This exploratory hypothesis-generating approach has been used by our group in several recent publications [30,33,34,35,36]. This information will set the foundation for future targeted studies with larger sample sizes to validate the untargeted results.

### 2.11. Identification and Annotation of Signals

The identification or annotation of peaks was performed through Progenesis QI by matching to an in-house physical standards library (consisting of approximately 2000 compounds run under the same instrument conditions as the study samples), as well as publicly available databases (HMDB, NIST, and METLIN), as previously reported [30,31]. Assignments were given based on matches to exact mass (MS), MS/MS fragmentation pattern, isotopic ion pattern, or retention time (RT, for in-house library standards only). Evidence for identification or annotation is denoted by an ontology system developed by our lab, as described previously [31,37]. In brief, OL1 refers to an in-house library match by MS, MS/MS, and RT; OL2a refers to an in-house library match by MS and RT; OL2b refers to an in-house library match by MS and MS/MS; PDa refers to a public database match by MS and experimental MS/MS (NIST and METLIN); PDb refers to a public database match by MS and theoretical MS/MS (HMDB); PDc refers to a public database match by MS and isotopic similarity); and PDd refers to a public database match by MS only.

### 2.12. Pathway Analysis of Untargeted Metabolomics Data

A pathway analysis was performed by using the MS Peaks to Pathways module in Metaboanalyst. All 6595 and 10,678 peaks remaining after preprocessing were used for liver and plasma analysis, respectively. Input files were generated for each two-way comparison and consisted of a four-column format: *m*/*z*, *p*-value, fold change, and retention time. A 5 ppm tolerance was used for liver samples, and a 3 ppm tolerance was used for plasma samples. A value of 0.05 was used for the *p*-value cutoff, and the Mus musculus (mouse) (KEGG) pathway library was used for the mummichog algorithm. Due to the exploratory nature of this study, *p*-value corrections for multiple comparisons were not performed.

## 3. Results and Discussion

### 3.1. Alterations in Dietary Folic Acid Leads to Changes in Body Weight in Mice

**Figure 2:**
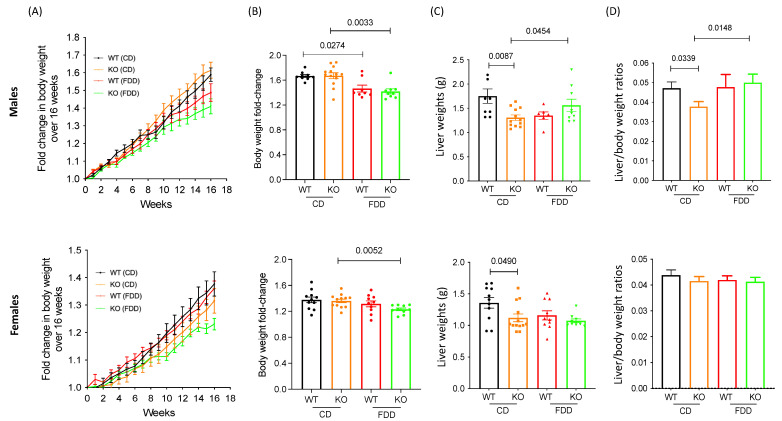

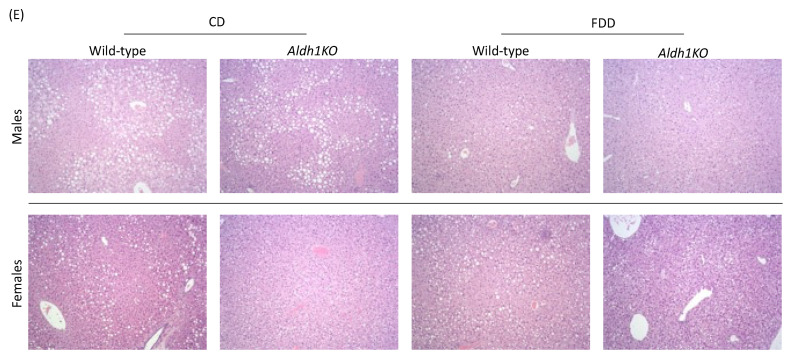
Comparison of body and liver weights and liver H&E between study groups. Body weights over 16 weeks (**A**) and corresponding fold change (**B**) of wild-type and *Aldh1l1* KO male (*upper panels*) and female (*lower panels*) mice fed control or folate-deficient diet. (**C**) Liver weight and (**D**) liver-to-body-weight ratio of male (*upper panels*) and female (*lower panels*) wild-type and *Aldh1l1* KO mice. (**E**) Representative H&E staining of livers from experimental groups. Data are represented as mean ± SEM, n = 9–12; *p* < 0.05 determined by Exact Wilcoxon Rank Sum Test was considered statistically significant. WT, wildtype; KO, *Aldh1l1* knockout; FDD, folate-deficient diet; CD, control diet.

The *Aldh1l1^+/+^* and *Aldh1l1^−/−^* littermates, both male and female, were randomly assigned to dietary groups at weaning. The body weights were recorded weekly, and the fold changes in body weights were calculated to account for the pre-diet weight variation (Figure 2A,B; data for male and female mice are shown). At the end of experiment, both *Aldh1l1^+/+^* and *Aldh1l1^−/−^* male mice gained less weight when fed an FD diet than their counterparts on control diet (*p* = 0.0274 and *p* = 0.0033, correspondingly). Similarly, the female *Aldh1l1^−/−^* mice on FD diet gained less weight than KO mice on the control diet (*p* = 0.0052). These changes are likely associated with the role of folate in providing carbon groups for biosynthetic reactions. Compared to male KO mice, females KO mice gained less weight (fold change 1.41 vs. 1.21) over a 16-week period. Interestingly, the *Aldh1l1^+/+^* females showed no significant difference in weight gain between the control and FD diets. Thus, the body-weight changes indicate that male and female mice gained weight differently on the same diet. We do not have a clear explanation for this phenomenon, but similar observations have been previously reported [27,38]. Of note, sexual dimorphism in metabolism, including one-carbon pathways, has been discussed in the literature [39,40].

### 3.2. Folate-Deficient Diet Affects Liver Histology in a Sex-Specific Manner

The liver weights of *Aldh1l1^−/−^* mice were lower than those for *Aldh1l1^+/+^* mice (*p* = 0.0087 for males and 0.0490 for females, correspondingly) (Figure 2C). These differences, however, can be attributed to the lower overall weight of these mice (Figure 2D; there are no differences between groups in females when the liver weight is adjusted for the body weight). The examination of the brain, lungs, kidneys, pancreas, heart, and spleen did not reveal any noticeable pattern in the organ size or weight between the genotypes, sexes and dietary groups (Supplementary Figures S2 and S3). The H&E analysis showed that livers of wild-type and *Aldh1l1* KO male mice develop lipid vacuolization in the organ which could be age related (the effect was observed in 6-month-old mice) (Figure 2E). The FDD caused a significant decrease in vacuolization in males for both genotypes, with a stronger effect in the KO male mice (Figure 2E). Vacuolization is also seen in CD-fed wild-type female mice (Figure 2E). In contrast to male mice, KO female mice on the control diet showed a significantly lower vacuolization when compared to WT female mice. In further contrast to male mice, vacuolization was not affected by dietary folate withdrawal in WT females (Figure 2E). Of note, in *Aldh1l1* KO female mice, the FDD caused a slight increase in liver vacuolization, the effect opposite to what was observed in KO male mice (Figure 2E). While the reason for such an effect is not clear, these results indicate a sex-specific effect of dietary folate on liver. Other organs did not show noticeable changes with diet and/or genotype (data not shown). 

### 3.3. Folate-Deficient Diet Leads to Hematological Changes in Aldh1l1^−/−^ Mice

**Figure 3:**
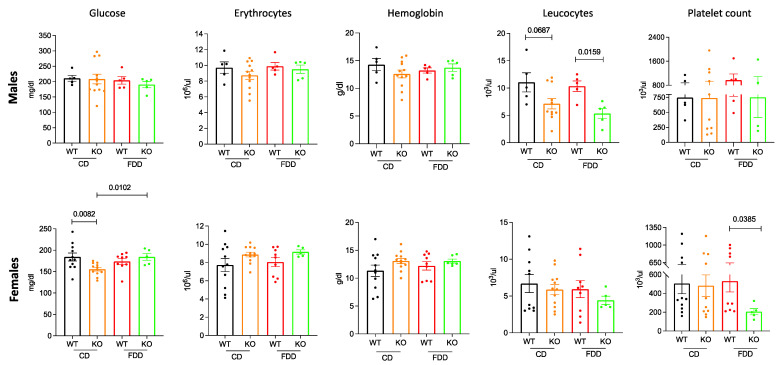
Whole-blood analysis showed alterations in glucose, erythrocyte count, hemoglobin, leucocyte count, and platelet count in *Aldh1l1^−/−^* and *Aldh1l1^+/+^* male (*upper panels*) and female (*lower panels*) mice on control and folate-deficient diet. White blood cell counts were significantly reduced in KO males regardless of diet, while, in females, the effect was seen only for those on the FD diet. Platelet counts were not different between genotypes on the control diet but were reduced in KO mice of both sexes on the FD diet. Data represented as mean ± SEM, n = 4–12. A *p* < 0.05 was considered statistically significant, determined by Exact Wilcoxon Rank Sum Test. WT, wild type; KO, *Aldh1l1* knockout; FDD, folate-deficient diet; CD, control diet.

The whole-blood analysis demonstrated that there was no major difference in blood glucose between WT and KO male mice on either diet (Figure 3). However, the KO females on control diet had lower blood glucose than their WT counterparts (*p* = 0.0082) (Figure 3). Surprisingly, glucose levels increased 1.17-fold in *Aldh1l1*^−/−^ female mice on the folate-deficient diet. Although not statistically significant, the erythrocytes or red blood cell (RBC) count was higher for WT males as compared to KO males in each diet (Figure 3). This trend was opposite for females (Figure 3). The results are particularly interesting in terms of sex differences irrespective of the genotype or diet. Hemoglobin measurements (Hb) also followed the same trends as the RBC counts, although the effects were not significant by genotype or diet (Figure 3). Interestingly, the leukocyte (white blood cell, WBC) counts were lower in *Aldh1l1*^−/−^ mice than *Aldh1l1*^+/+^ for both sexes (significant for males, *p* = 0.0687 on CD and *p* = 0.0159 on FDD). The count was lower in the FDD group, thus showing that the low folate diet exacerbated the lowering of WBC in KO mice, particularly in males. Lower WBC counts in *Aldh1l1^−/−^* mice can be due to an altered inflammatory profile, as we reported previously [20]. Moreover, the phenomenon that low dietary folate can result in reduced peripheral leucocyte number has been reported [41]. It is not clear whether this was a targeted effect on these cells or more the reflection of a systemic effect. Male mice did not show any difference in the platelet count on both diets, and this can be due to the high variability among animals in each group (Figure 3). *Aldh1l1*^−/−^ female mice showed a statistically significant lower platelet count (*p* = 0.0385) than *Aldh1l1*^+/+^ mice when fed a folate-deficient diet. Taken together, these observations indicate that mice kept on CD and FDD for a 16-week period maintained a normal physical phenotype regardless of sex or genotype but exhibited differences in several blood parameters with respect to sex, genotype, and diet. 

The literature underscores ALDH1L1 as a main regulator of folate metabolism [9,42]. In support of this role, we have previously reported that the knockout of the enzyme in mice produces a metabotype indicative of functional folate deficiency, though no apparent phenotypic features or histological changes were found in the KO mice [20]. In the present study, we combined *Aldh1l1* KO and dietary folate restriction with the expectation that such a diet might exacerbate the phenotype and metabotype associated with the ALDH1L1 function. Of note, the combination of the KO and folate-deficient diet resulted in decreased body weight in both male and female mice (Figure 2). Also, differences in liver histology were noted with decreased vacuolization in KO female mice fed a standard diet and in both male genotypes fed a folate-deficient diet (Figure 2). Furthermore, the weight of livers was different between WT and KO groups on the control diet, but this was not the case when mice were fed a folate-deficient diet. Moreover, there were no differences between corresponding groups in females when the liver weight was adjusted for the body weight. In our previous study, mice were fed the diet with 4% fat. However, to match the folate-deficient diet, in the present study, the control diet contained 6% fat. Thus, the control diet in the present study was softer and more palatable, and this could be the cause of the additional effects observed in the present study, especially taking into consideration that the present study was run over a relatively long diet exposure (16 weeks). Of note, our data are in line with previous findings that low dietary folic acid supplementation leads to hematopoietic changes [41]. To clarify whether hematopoietic changes observed in our study are linked to the ALDH1L1 function would require additional studies.

It is important to emphasize that, though mice were fed a folate-deficient diet in our study, numerous reports indicate that such a diet is not sufficient to induce severe folate deficiency in rodents, since they also obtain folate from intestinal bacteria [43,44]. To prevent this route of the folate supply, mice should be given an antibiotic to kill gut bacteria [45]. Since our goal was not to study severe folate deficiency but, rather, to explore the interaction of ALDH1L1 with the diet, we did not include antibiotics in these experiments. ALDH1L1 protein is most abundantly expressed in the liver, the main organ of folate and amino acid metabolism, and our previous study showed that *Aldh1l1* KO produces a strong effect on liver metabotype [20]. Therefore, in this study, we focused on the liver metabolic profiles as well, but also evaluated how metabolic changes in the liver were reflected in plasma. 

### 3.4. Overall Metabolomic Analysis

After the preprocessing of the untargeted metabolomics data, 6595 and 10,678 features for the liver and plasma datasets, respectively, were subjected for further analysis. PCA plots of the preprocessed liver (Supplementary Figure S1A) and plasma (Supplementary Figure S1B) datasets showed tight clustering of total QCSPs in relation to the total variation of the datasets, indicating that the datasets were of sufficient quality and the data preprocessing steps were appropriate [46]. 

**Figure 4:**
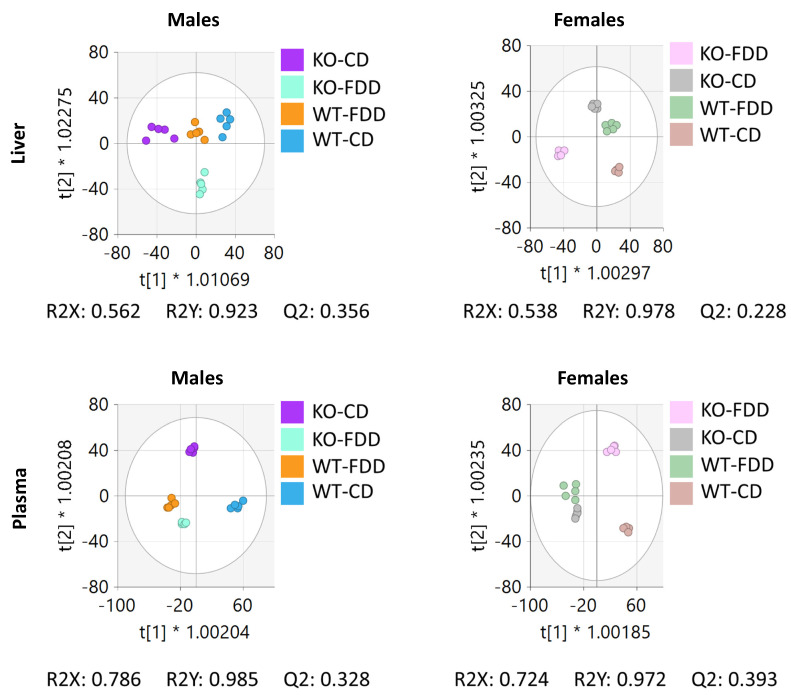
OPLS-DA shows that Aldh1l1 KO mice have the metabotype more similar to the metabotype of WT mice kept on a folate-deficient diet. OPLS-DA plots were built by using the untargeted liver data for all males and females (*upper panels*) to visualize separations based on *Aldh1l1* KO and/or dietary folate deficiency. Similarly, OPLS-DA plots were built by using the untargeted plasma data for all males and females (*lower panels*). For both liver and plasma groups, n = 4–5.

OPLS-DA, a supervised multivariate technique [47], was used to determine the variable importance to projection (VIP) of peaks for pairwise comparisons by sex, genotype, and diet. The OPLS-DA visualizations for the liver and plasma samples of males and females are shown in Figure 4. The clear separation of each group indicates that genotype and diet, alone or in combination, gave distinct metabolic profiles in both types of biospecimens. The model statistics for the pairwise comparisons by sex, genotype, and diet are provided in Table 1. All comparisons showed strong model statistics (R2X > 0.5; R2Y > 0.5; Q2 > 0.5, with the exception of the CD vs. FDD comparison in KO females for plasma samples) for each pairwise comparison, indicating that dietary folate and *Aldh1l1* alone or in combination have a significant effect on the liver and plasma metabolomes of male and female mice. 

To expand upon our connection of multivariate results with our univariate results, we performed a sparse PLS-DA (sPLS-DA) analysis of liver and plasma samples based on sex, genotype, and diet (Supplementary File S2). The loadings plots revealed metabolite signatures associated with separating samples by each parameter (male vs. female, WT vs. KO, or CD vs. FD). Of note, sex was the strongest contributor in the group separation, the phenomenon especially apparent in the liver (Supplementary File S2). These analyses allowed us to identify metabolite classes, which represent specific pathways further analyzed in the univariate analysis below.

### 3.5. Metabolic Pathway Perturbations Due to Aldh1l1 KO and Dietary Folate Restriction 

To gain a network-level view of metabolic processes that are affected by *Aldh1l1* KO and/or dietary folate restriction, we performed pathway analyses for all pairwise comparisons for both liver and plasma, using MetaboAnalyst. For liver samples, pathways involved in carbohydrate, amino acid, and lipid metabolism were altered in response to KO status and/or folate restriction (Supplementary Table S2). For males, the greatest number of pathway perturbations was seen when comparing CD and FDD for the Aldh1l1 KO mice. Similarly, this comparison was tied for the greatest number of pathway perturbations in female mice, although the specific pathways were highly different between the two sexes. Additionally, female mice had a higher number of altered pathways than male mice when compared the WT genotype fed CD vs. FDD. A similar extent of the sex-specific effect was observed when two genotypes fed the FD diet were compared (Supplementary Table S2). These results indicate that male and female mice have distinct hepatic metabolic responses to *Aldh1l1* KO and/or folate restriction, with females seemingly having a broader number of affected metabolic pathways. This suggests that hepatic metabolic responses to *Aldh1l1* KO and folate restriction are not only highly complex but also sex specific. 

The pathway analysis of plasma samples also showed perturbations in carbohydrate, amino acid, and lipid metabolism (Supplementary Table S3). Interestingly, males had a higher number of perturbed pathways compared to females in all but one pairwise comparison. Thus, the plasma metabolome of male mice shows a broader number of affected metabolic pathways as compared to females, in contrast to what was observed for the liver samples. This suggests that male mice have a stronger systemic response to the *Aldh1l1* loss and/or folate restriction, while female mice have a more localized effect to the liver. In agreement with findings from the liver metabolome analysis, the types of perturbed metabolic pathways between each comparison in plasma are highly diverse and varied greatly between the two sexes. This also suggests that, in response to *Aldh1l1* KO and folate restriction, the plasma metabolome undergoes highly diverse and sex-specific changes, a phenomenon also observed in the liver. Of note, sex-specific effects on metabolomic profiles of both gene knockouts and the diet composition, including folate content, have been reported [26,27,28].

### 3.6. Overall Effect of the Genotype and Diet

**Figure 5:**
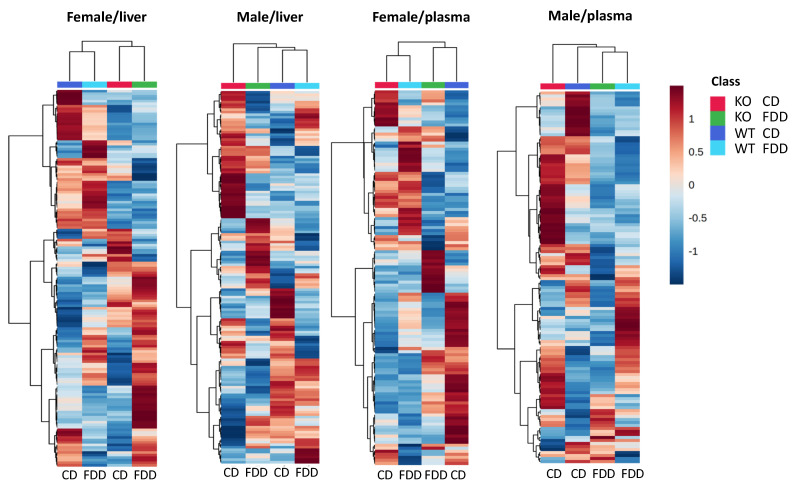
Differential perturbation of metabolite pathways by *Aldh1l1* knockout and a folate-deficient diet between males and females. Heatmaps showing comparison of liver and plasma samples from WT and KO female and male mice fed a control or folate-deficient diet. Metabolites included in heatmaps are those that satisfy the criteria of OL1 or OL2a ontology levels. Heatmaps are auto-scaled (mean-centered and divided by standard deviation) for each variable; n = 4–5 per group.

Our study indicates that the main discriminator between metabotypes of studied groups is sex with genotype as the second contributing factor, while the effect of a folate-deficient diet was less prominent (Supplementary Tables S2 and S3). These conclusions were based on 6595 and 10,678 peaks for liver and plasma samples, respectively, most of which represent unknowns. The sex-specific metabolic profiles, as well as differential responses of metabolites to certain conditions/treatments in males and females, were noted in several studies [25,27,48,49,50,51,52]. To gain a better understanding of the metabolic effects of *Aldh1l1* KO and dietary folate restriction, we performed a more detailed analysis based on our most confidently identified metabolites in the liver and plasma (OL1 and OL2a ontology level). To understand how the profiles of these metabolites were affected by genotype and diet, heatmaps were generated for each sex for liver and plasma samples (Figure 5). The overall comparison between groups based on these metabolites confirmed the conclusion that sex is the main metabotype discriminator for both liver and plasma, as seen by the differences in heatmap profiles between males and females, as genotype and diet are altered (Supplementary Files S3 and S4). This analysis also highlighted metabolites that were most different between groups for each sex which are explored in more detail below.

### 3.7. Glycine and Glycine Conjugates

**Figure 6:**
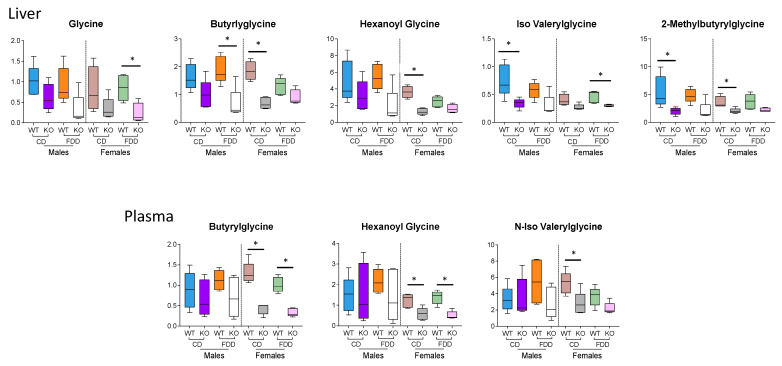
Concentrations of glycine and glycine conjugates were reduced in both liver and plasma of *Aldh1l1* KO mice. Metabolites were identified by using the untargeted data at a level of OL1 or OL2a; n = 4–5 per group; * *p* < 0.05.

As we previously reported, one of the most profound metabolic effects in the liver of *Aldh1l1* KO mice was a decrease of glycine and several glycine conjugates [20]. A similar effect was observed in the current study, as well (Figure 6). Of note, dietary folate withdrawal neither produced such an effect nor modified the effect of the ALDH1L1 loss. It appears that the genotype-associated effect on glycine and its conjugates took place in both male and female mice to a similar extent. In the present study, the decrease of several glycine conjugates was also observed in the plasma of *Aldh1l1* KO mice (Figure 6).

### 3.8. Acylcarnitines

**Figure 7:**
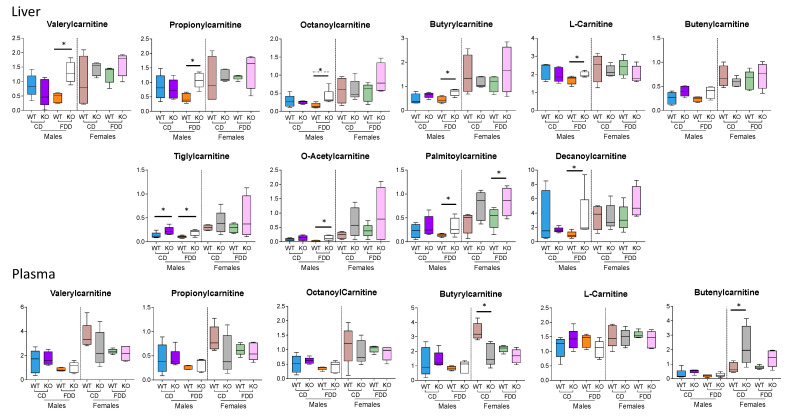
Liver and plasma acylcarnitines are generally higher in female than in male mice. Metabolites were identified by using the untargeted data at a level of OL1 or OL2a; n = 4–5 per group for liver and plasma; * *p* < 0.05.

Acylcarnitines contributed significantly to the differentiation of the metabotype between male and female mice (Figure 7). In general, levels of these metabolites were higher in both the liver and plasma of female mice as compared to male mice. This effect was especially noticeable for WT mice fed the control diet and was observed in both the liver and plasma. Moreover, hepatic levels of acylcarnitines in female mice generally did not show significant differences between genotypes or diets, while male mice showed significant increases in multiple acylcarnitine species when comparing WT and KO mice on a folate-deficient diet. This difference was largely due to lower levels of acylcarnitine species in WT males on a folate-deficient diet. Acylcarnitines are intermediates of fatty acid oxidation, and with folate deficiency, they have been shown to lead to the impairment of fatty acid oxidation [53,54]. Such a link could explain generally lower levels of acylcarnitines in the FDD group. Interestingly, further disruption of folate metabolism through the loss of *Aldh1l1* increased acylcarnitine species in male mice, suggesting that the combination of the KO with the folate-deficient diet counteracted this perturbation. While not statistically significant, this trend was also observed in females. Taken together with the histological data (Figure 2), these results indicate that *Aldh1l1* KO and dietary folate deficiency alter hepatic lipid/fatty acid metabolism in mice, with males showing a stronger response as compared to females. Of note, plasma acylcarnitine levels showed fewer significant alterations than acylcarnitine levels in the liver, with concentrations in female mice being generally higher than in male mice, though statistical significance was not reached when comparing these groups. Additionally, KO female mice on a control diet showed higher concentrations of several plasma acylcarnitines compared to their WT counterparts. This indicates that female mice may have stronger alterations in systemic acylcarnitine profiles compared to males, and these differences are largely seen only when fed a control diet. 

### 3.9. Folate-Related Metabolites

**Figure 8:**
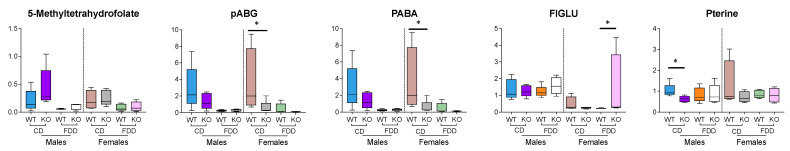
*Aldh1l1* KO increased liver 5-methyl-THF only in males, while the FD diet reduced this form of folate in both sexes independent of genotype. Metabolites were identified by using the untargeted data at a level of OL1 or OL2a; n = 4–5 per group; * *p* < 0.05.

As would be expected upon folic acid withdrawal from the diet, levels of 5-methyltetrahydrofolate (5-methyl-THF), the main form of folate in the liver, were decreased in all groups on the folate-deficient diet compared to the corresponding groups on the control diet (Figure 8). In these experiments, however, we can assign only the monoglutamate species that represent a fraction of cellular folates. In agreement with the folate intake, much higher levels of glutamylated PABA (pABG), the product of folate degradation [55], were seen in liver of both male and female animals fed the control diet (Figure 8). Of note, the loss of *Aldh1l1* results in the decrease of pABG in liver, which is similar to the effect of dietary folate withdrawal. Further, in the liver, levels of para-aminobenzoic acid (PABA) mirror the pABG levels in both male and female mice (Figure 8), suggesting that the presence of this metabolite is likely associated with the further hydrolysis of pABG. While such a reaction was not reported in humans, the enzyme hydrolyzing pABG is present in plants and bacteria [56,57]. Folate degradation also produces pterin [55], which was higher in the livers of WT male mice fed the control diet as compared to the KO mice on the CD, but this effect was not seen in female mice. A less obvious link between dietary folate and pterin (Figure 8) could be associated with further oxidation of this metabolite in the liver [58]. The elevation of FIGLU, a marker of folate deficiency, overall was not observed. However, the combination of the *Aldh1l1* KO and folate-deficient diet produced a significant FIGLU increase in female mice, indicating a more severe folate deficiency (Figure 8). Further, these data are in agreement with previous studies which show that, by itself, the withdrawal of dietary folate in mice is not sufficient to induce folate deficiency [43,44].

### 3.10. Methylation-Related Metabolites

**Figure 9:**
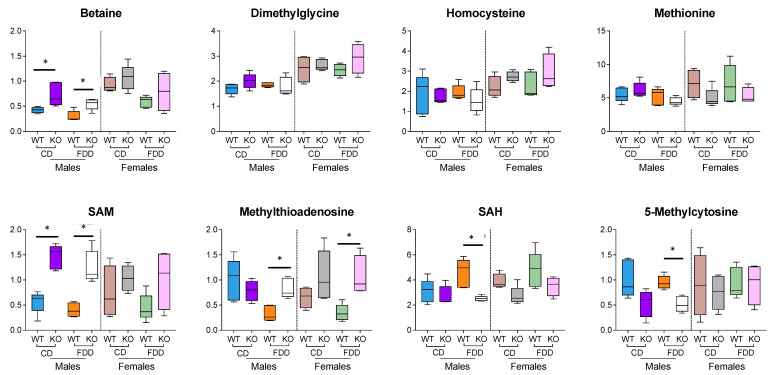
Methyl group donors in the liver were not affected by FA deficiency but were elevated in both KO males and KO females. Metabolites were identified by using the untargeted data at a level of OL1 or OL2a; n = 4–5 per group; * *p* < 0.05.

Folate, in the form of 5-methyl-THF, is involved in the re-methylation of homocysteine to methionine. The major portion of cellular methionine is consumed in the biosynthesis of SAM, the key metabolite for methylation reactions. These reactions include (i) methylation of large biomolecules (DNA, RNA and proteins) [59]; (ii) biosynthesis of polyamines [60]; (iii) biosynthesis of creatine [61]; and (iv) other small molecule biosynthesis [62] (Figure 1). Interestingly, levels of SAM in the liver of KO male mice were significantly higher compared to wild-type male mice (Figure 9). This effect was not changed upon folate withdrawal and was observed in both sexes; however, only male mice showed statistical significance for this effect. We interpret these changes as a decrease in the use of SAM for methylation processes not associated with polyamine and creatine biosynthesis. In agreement with this assessment, the levels of S-adenosylhomocysteine (SAH) were decreased in the KO mice in both sexes, particularly for male mice, on the FDD (Figure 9). For the folate-deficient diet, levels of methylthioadenosine (MTA) were significantly elevated in KO compared to WT mice for both sexes (Figure 9), suggesting an increased flow of SAM toward polyamine biosynthesis in KO mice. Of interest is the observation that levels of methionine were not noticeably affected by the withdrawal of folic acid from the diet (Figure 9). This phenomenon can be explained by the alternative pathway of homocysteine re-methylation, the reaction converting betaine to dimethylglycine (Figure 1), or by preserving methyl groups by limiting the overall methylation of macromolecules. Interestingly, increased levels of betaine were observed in the livers of male KO mice on either diet, supporting increased flux through this methionine-salvaging pathway. Overall, these effects were more pronounced in male mice as compared to female mice, indicating a sex-specific role for these pathways.

**Figure 10:**
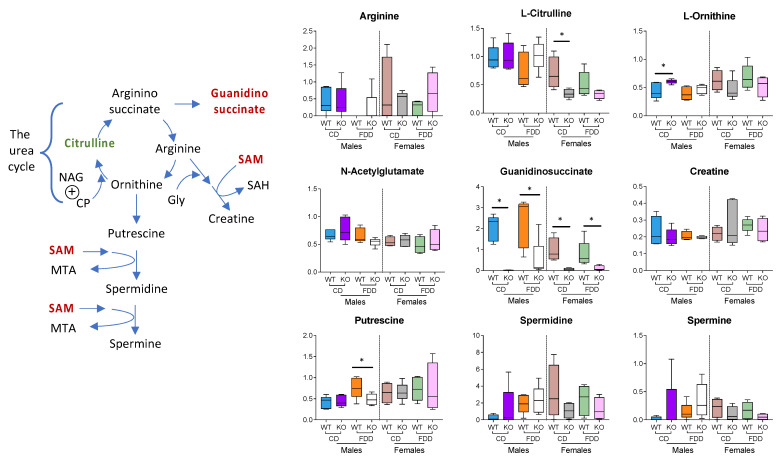
The urea cycle metabolites L-citrulline and guanidinosuccinate differ in mouse livers depending on sex and genotype. Metabolites were identified by using the untargeted data at a level of OL1 or OL2a; n = 4–5/group; * *p* < 0.05. Schematic on the *left* describes links between the urea cycle and polyamine and creatine biosynthesis (NAG, N-acetylglutamate; CP, carbamoyl phosphate; MTA, methylthioadenosine; SAM, S-adenosyl-L-methionine; NAG is an activator of carbamoyl phosphate synthetase, which is indicated by *circled plus*-sign).

Our observation of the link between ALDH1L1 and methylation-related metabolites in the liver is in line with previously reported regulation of the flux of methyl groups by the enzyme in glioblastoma cells [63]. Interestingly, a strong drop of guanidinosuccinate in KO mice of both sexes despite the composition of the diet also indicates the involvement of ALDH1L1 in the regulation of methylation fluxes (Figure 10). Previously, it has been shown that guanidinosuccinate declines sharply in the serum and urine of animals and humans upon high intake of methionine [64]. On a more general note, our data indicate the link between folate metabolism and the urea cycle (Figure 10, schematic). The urea cycle provides metabolites for both polyamine and creatine biosynthesis (ornithine and arginine, respectively), with the liver being the only organ where this cycle is active. KO male mice on the control diet showed increased levels of ornithine, while KO female mice on this diet showed a decrease in citrulline. Though these effects were not statistically significant in the animals fed a folate-deficient diet, the overall trend remained. 

## 4. Conclusions

Overall, though the *Aldh1l1* KO produces metabolic effects that are indicative of functional folate deficiency, the dietary folate restriction itself does not fully recapitulate the metabolic effect of the KO. Furthermore, the ALDH1L1 loss apparently has a stronger effect on liver metabolism than dietary folate withdrawal. Interestingly, dietary folate withdrawal enhanced the metabolic perturbations linked to the ALDH1L1 loss only for a subset of metabolites, such as acylcarnitines. Importantly, both the ALDH1L1 loss and dietary folate deficiency produced different metabolic effects depending on sex. Therefore, dietary folate recommendations should employ a precision nutrition approach that requires consideration of both genotype and gender. 

## Figures and Tables

**Table 1:** Summary of OPLS-DA model statistics for liver and plasma pairwise comparisons. OPLS-DA model statistics (R2X, R2Y, and Q2) were calculated for each pairwise comparison in SIMCA 15.

**Liver**				
	**Groups**	**R2X**	**R2Y**	**Q2**
*Males*				
	WT-CD vs. FD	0.721	1	0.749
	KO-CD vs. FD	0.783	1	0.755
	FDD-WT vs. KO	0.814	1	0.551
	CD-WT vs. KO	0.777	1	0.841
*Females*				
	WT-CD vs. FD	0.853	1	0.547
	KO-CD vs. FD	0.855	1	0.81
	FDD-WT vs. KO	0.833	1	0.748
	CD-WT vs. KO	0.864	1	0.614
**Plasma**				
	**Groups**	**R2X**	**R2Y**	**Q2**
*Males*				
	WT-CD vs. FD	0.882	1	0.924
	KO-CD vs. FD	0.914	1	0.506
	FDD-WT vs. KO	0.883	1	0.689
	CD-WT vs. KO	0.853	1	0.624
*Females*				
	WT-CD vs. FD	0.814	1	0.762
	KO-CD vs. FD	0.319	0.914	0.384
	FDD-WT vs. KO	0.689	1	0.78
	CD-WT vs. KO	0.898	1	0.834

## Data Availability

All data are included in the paper and the Supplementary Materials.

## Supplementary Materials

The following supporting information can be downloaded at: https://www.mdpi.com/article/10.3390/metabo12050454/s1. Supplementary File S1 figure legends: Supplementary Figure S1. PCA analyses of all liver (A) or plasma (B) datasets and their corresponding QCSPs show sufficient quality of the datasets and appropriate pre-processing of the data. Supplementary Figure S2. No patterns in organs weights could be seen due to genotype, sex, or diet. FDD—folate-deficient diet; CD—control diet. Data represent mean ± SEM, n = 6–12; a *p* < 0.05 was considered statistically significant, determined by the Exact Wilcoxon Rank Sum Test. Supplementary Figure S3. No patterns in organ/body weight ratios could be found due to genotype, sex, or diet. FDD—folate-deficient diet; CD—control diet. Data represent mean ± SEM, n = 6–12. A *p* < 0.05 was considered statistically significant, determined by Exact Wilcoxon Rank Sum Test. Supplementary Table S1 (included in the Supplementary File S1): Primers used for genotyping mice for wild-type or mutant allele. Supplementary File S2 figure legends: Supplementary Figure S4. sPLS-DA plots and loading scores for liver samples. Samples were classified as WT or KO, male or female, CD or FDD, or all three. Supplementary Figure S5. PCA plots of liver samples using all preprocessed signals, signals matched by OL1 or OL2a, or metabolites selected by the sPLS-DA analysis. Supplementary Figure S6. Heatmap of sPLS-DA-selected metabolites across all groups for liver. Supplementary Figure S7. sPLS-DA plots and loading scores for plasma samples. Samples were classified as WT or KO, male or female, CD or FDD, or all three. Supplementary Figure S8. PCA plots of plasma samples using all preprocessed signals, signals matched by OL1 or OL2a, or metabolites selected by the sPLS-DA analysis. Supplementary Figure S9. Heatmap of sPLS-DA-selected metabolites across all groups for plasma. In all cases, n = 4–5 per group for the liver and 5 per group for plasma. Supplementary File S3: Detailed heatmap showing metabolites differences in male and female mice liver in all 8 groups. Supplementary File S4: Detailed heatmap showing metabolites differences in male and female mice plasma in all 8 groups. Supplementary Table S2: Table showing pathways with *p*-value < 0.1 for liver Sample Pairwise Comparisons. Pathway analysis was performed by using MetaboAnalyst. The *p*-values are only displayed in instances where *p* < 0.1. Supplementary Table S3: Table showing pathways with *p*-value < 0.1 for plasma Sample Pairwise Comparisons. Pathway analysis was performed by using MetaboAnalyst. The *p*-values are only displayed in instances where *p* < 0.1.
