# Supplementary material for: Sex-Specific Metabolic Effects of Dietary Folate Withdrawal in Wild-Type and Aldh1l1 Knockout Mice
Source: Metabolites. 2022 May 18;12(5):454. doi: 10.3390/metabo12050454 (PMC9143804; doi:10.3390/metabo12050454)
Supplement: Supplementary file 1 [file metabolites-12-00454-s001.zip › Supplementary file S1.pdf]

Supplemental Figure S1

(A)

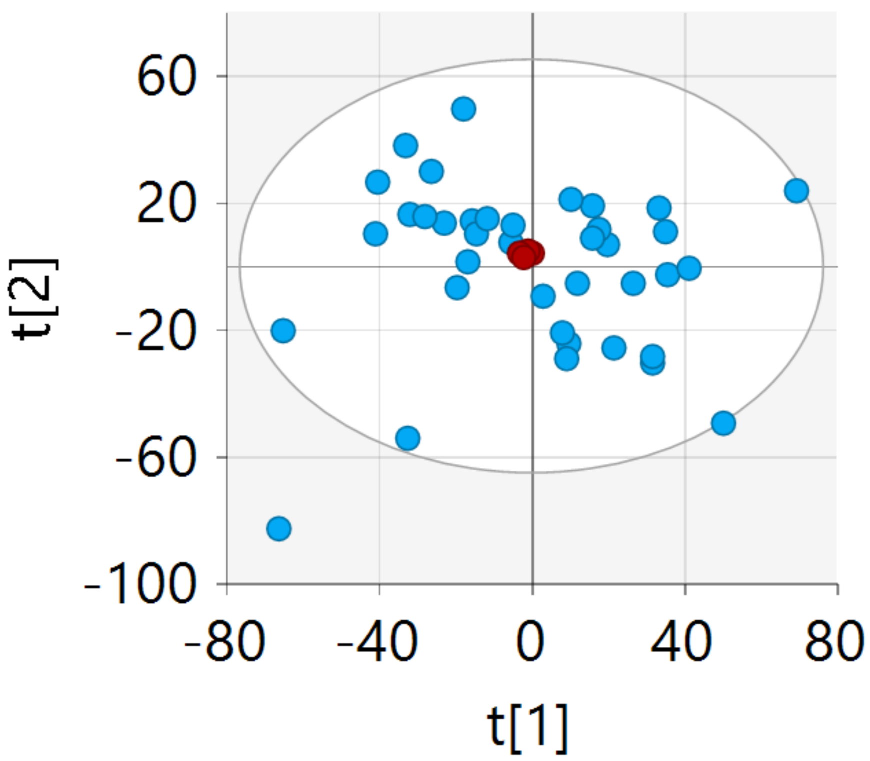

R2X: 0.604

(B)

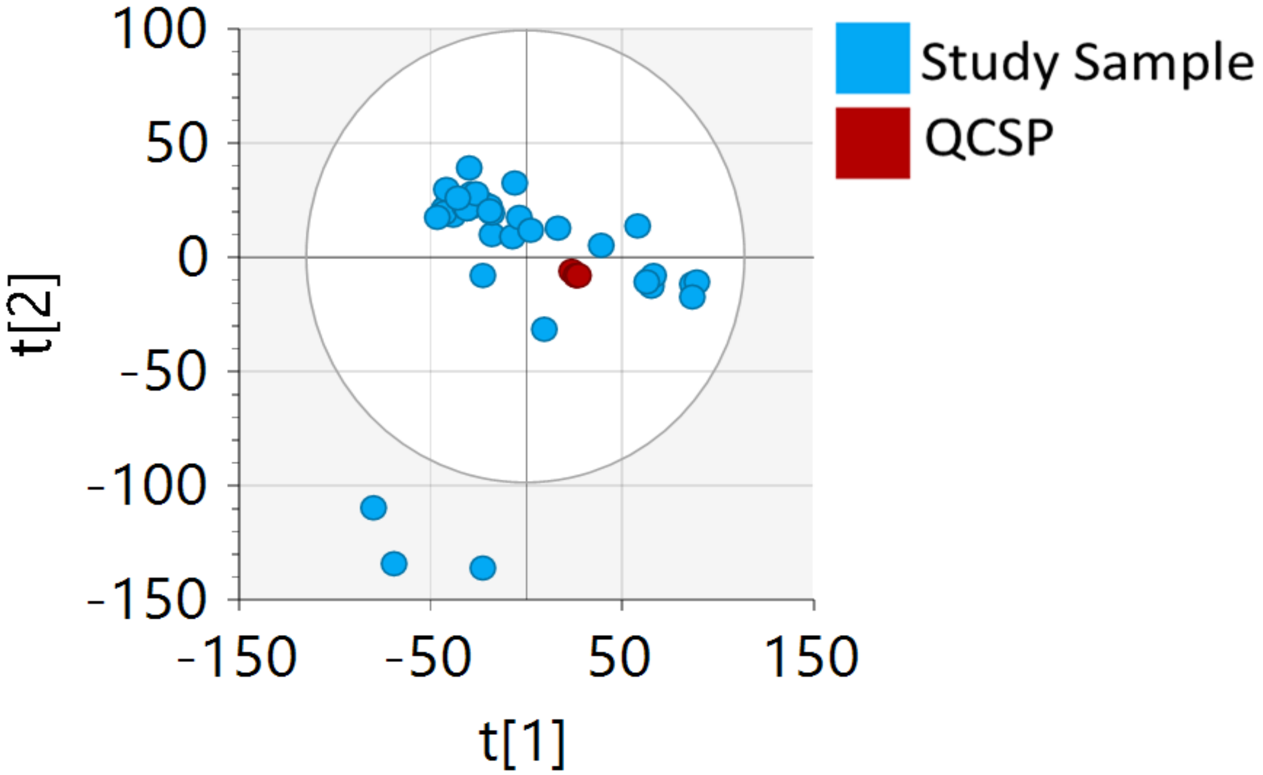

R2X: 0.604

Supplemental Figure S2

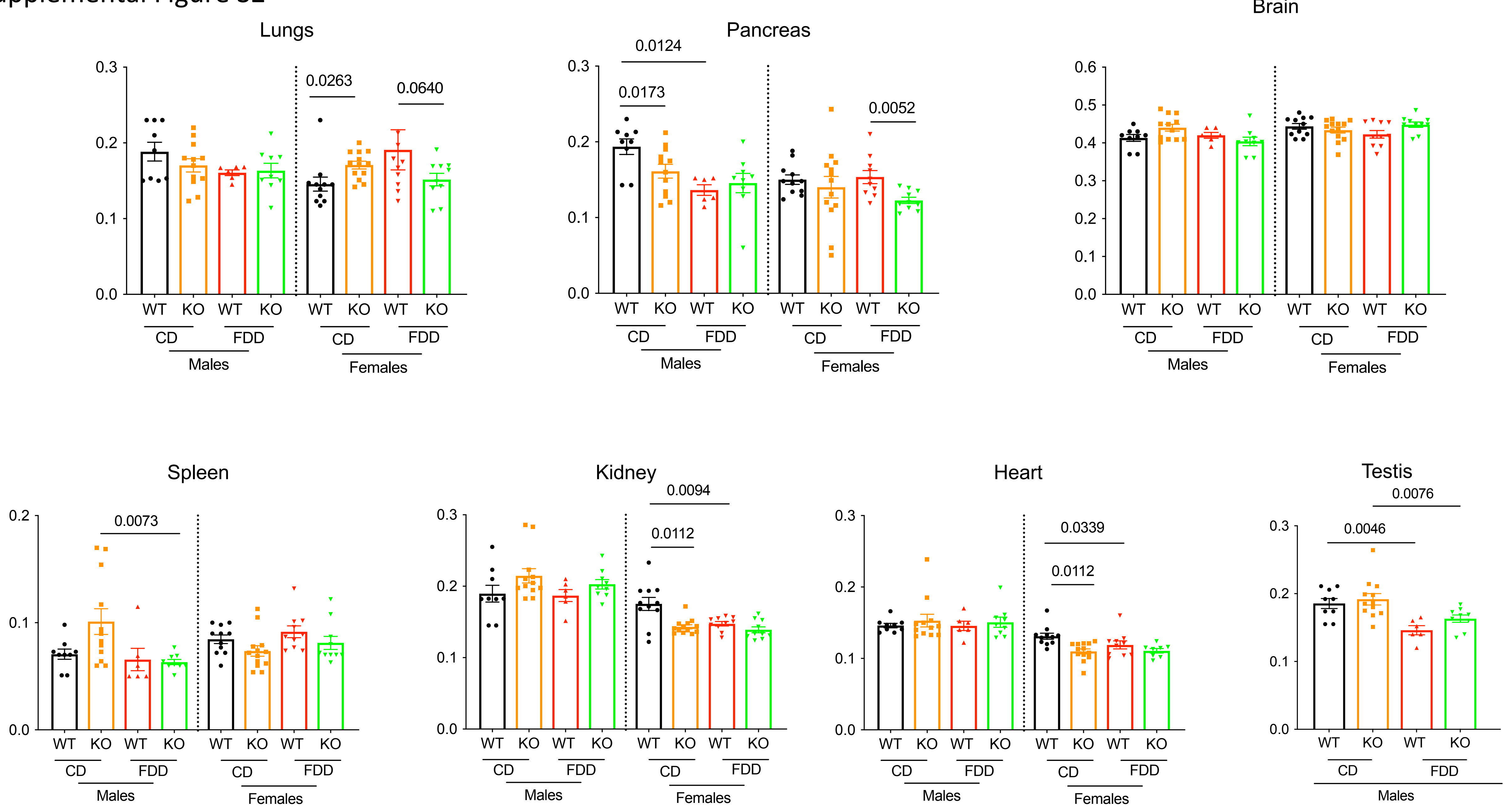

Supplemental Figure S3

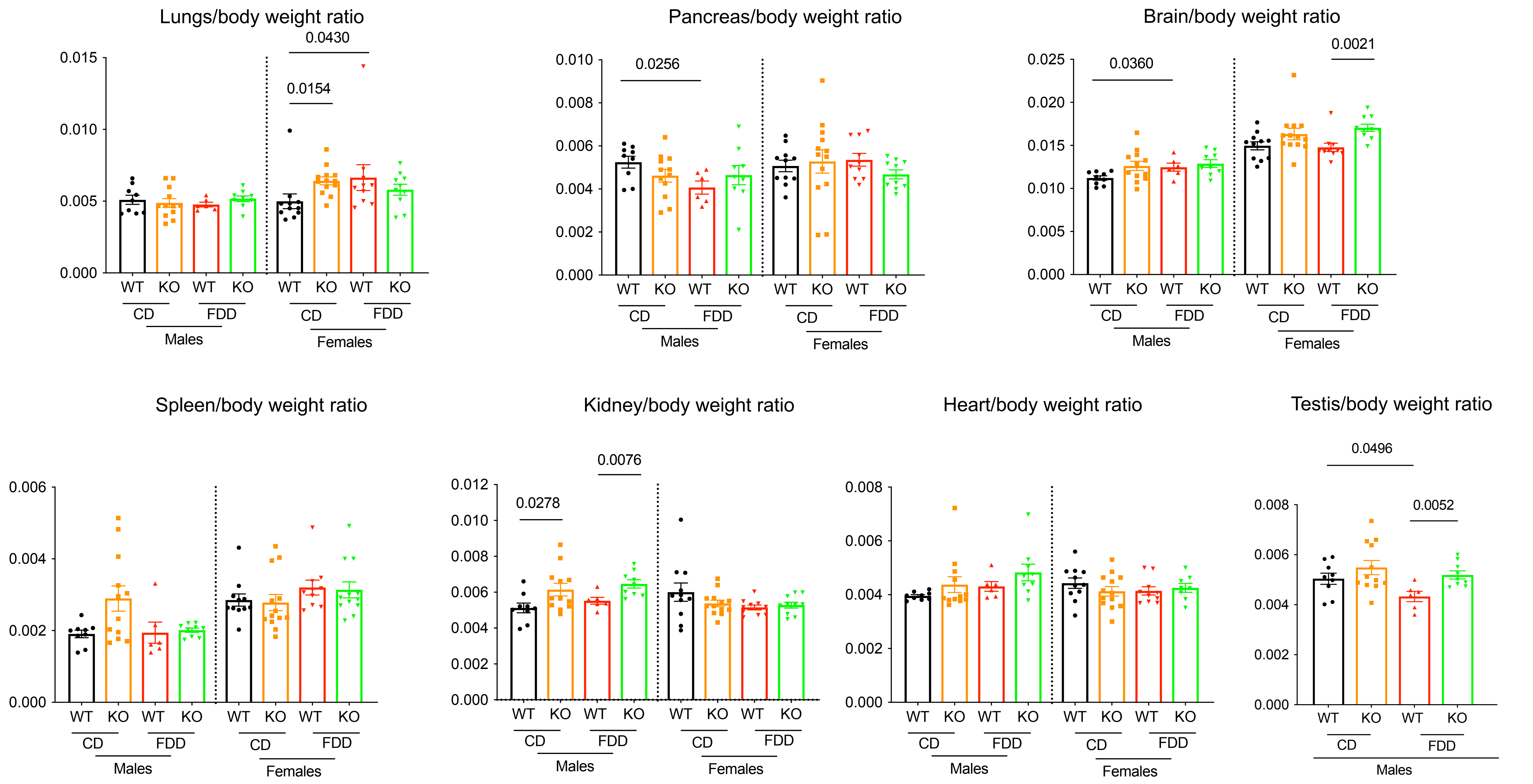

Supplemental Table S1:

Primers	Sequence	Size(bp)
**WT-F**	5′- CCGAAGGACCAAACTCTTTCC-3′	199 bp
**WT-R**	5′- TCCTTACAGCCCTTTCCAGAG-3′	
**Mut-F**	5′- CACACCTCCCCCTGAACCTGAAA -3′	685 bp
**Mut-R**	5′- AAGCTTCCTCTCTGTTCTACCCACAC-3′	
