# Supplementary material for: Sex-Specific Metabolic Effects of Dietary Folate Withdrawal in Wild-Type and Aldh1l1 Knockout Mice
Source: Metabolites. 2022 May 18;12(5):454. doi: 10.3390/metabo12050454 (PMC9143804; doi:10.3390/metabo12050454)

Supplementary Figure S4

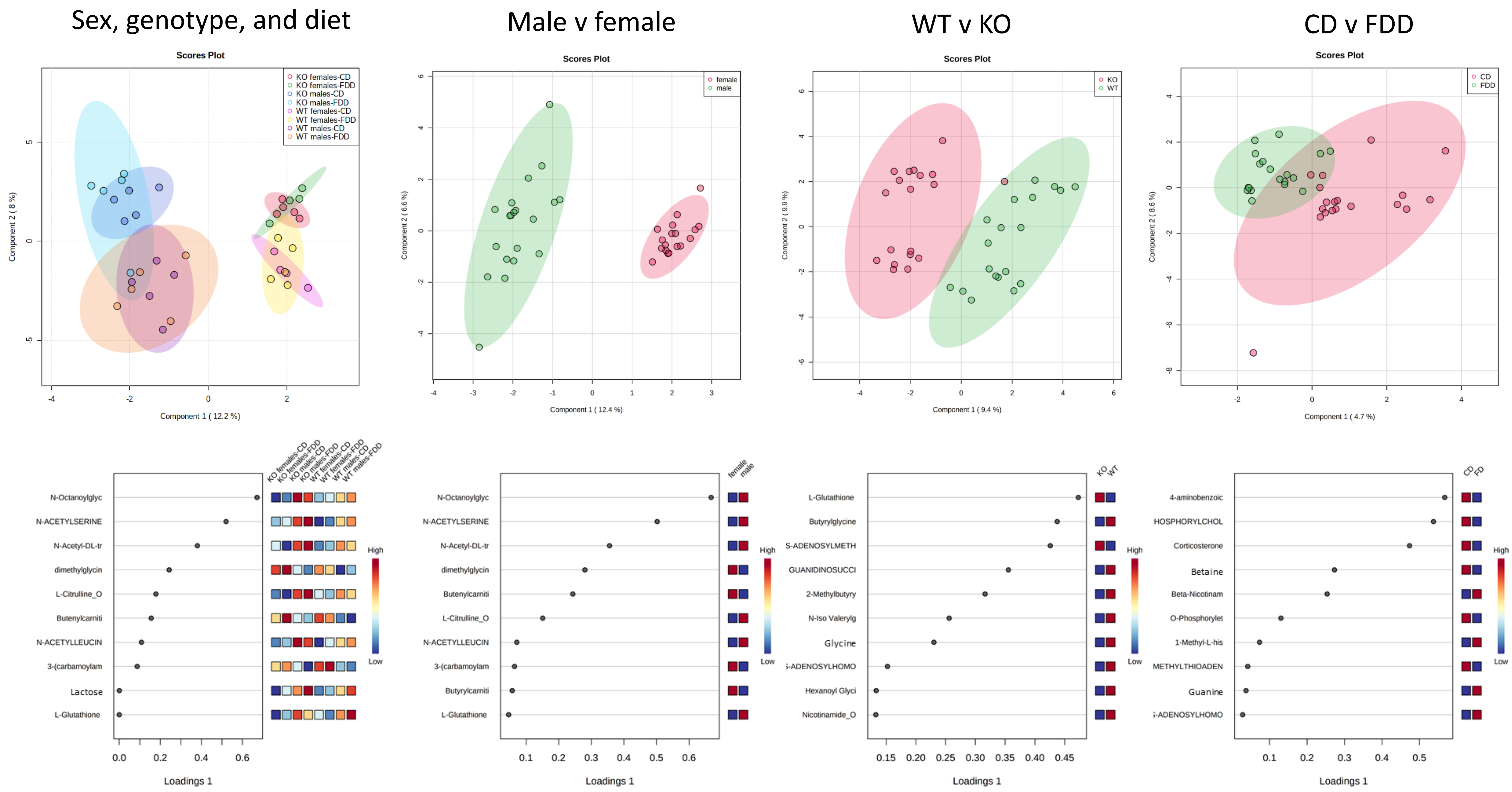

Supplementary Figure S5

All peaks

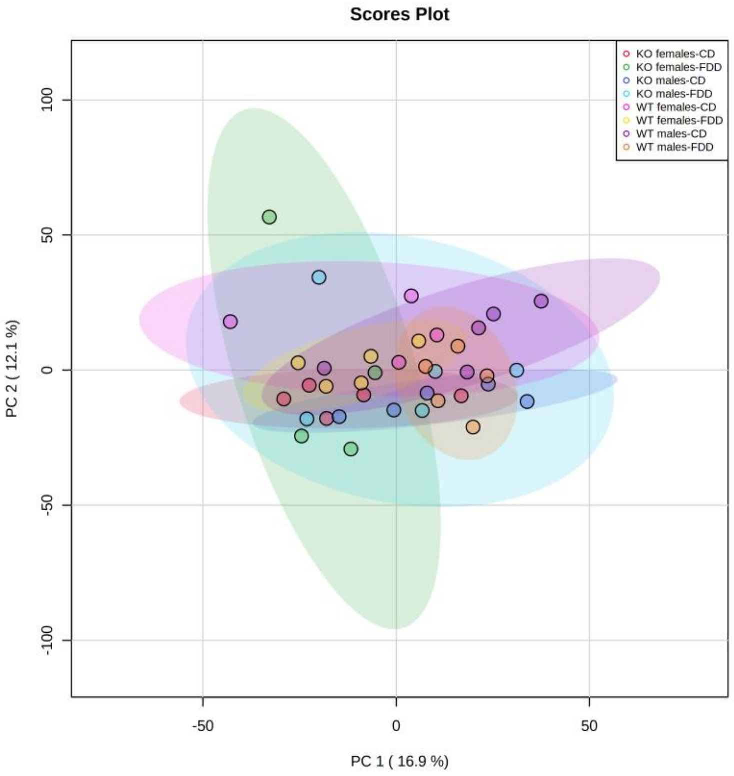

Identified Metabolites (OL1/OL2a)

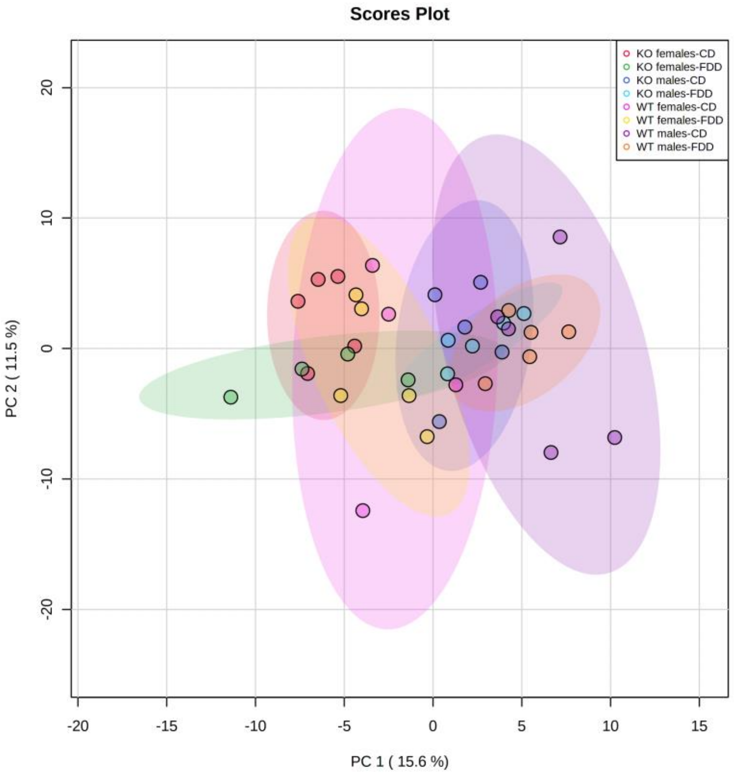

sPLS-DA selected metabolites

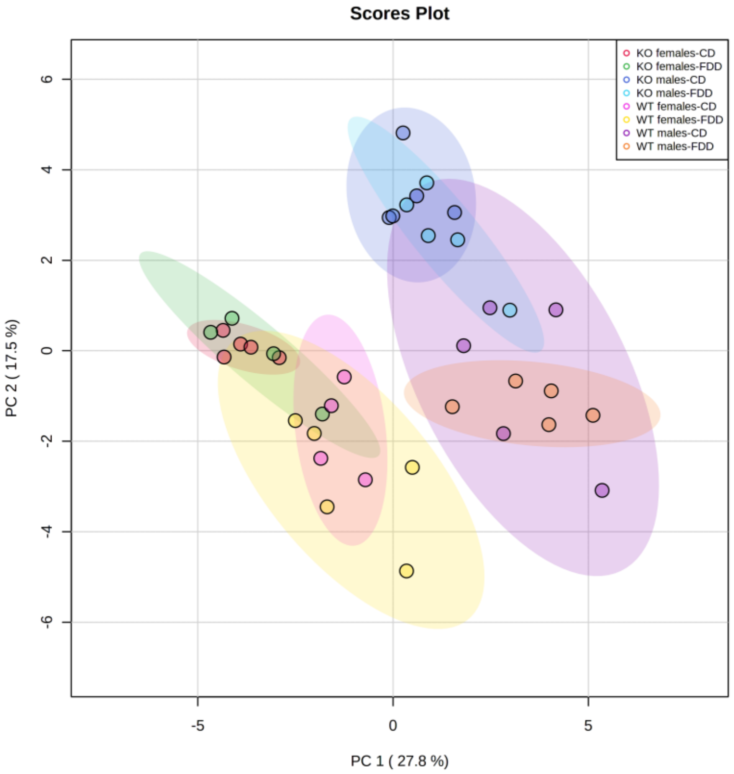

Supplementary Figure S6

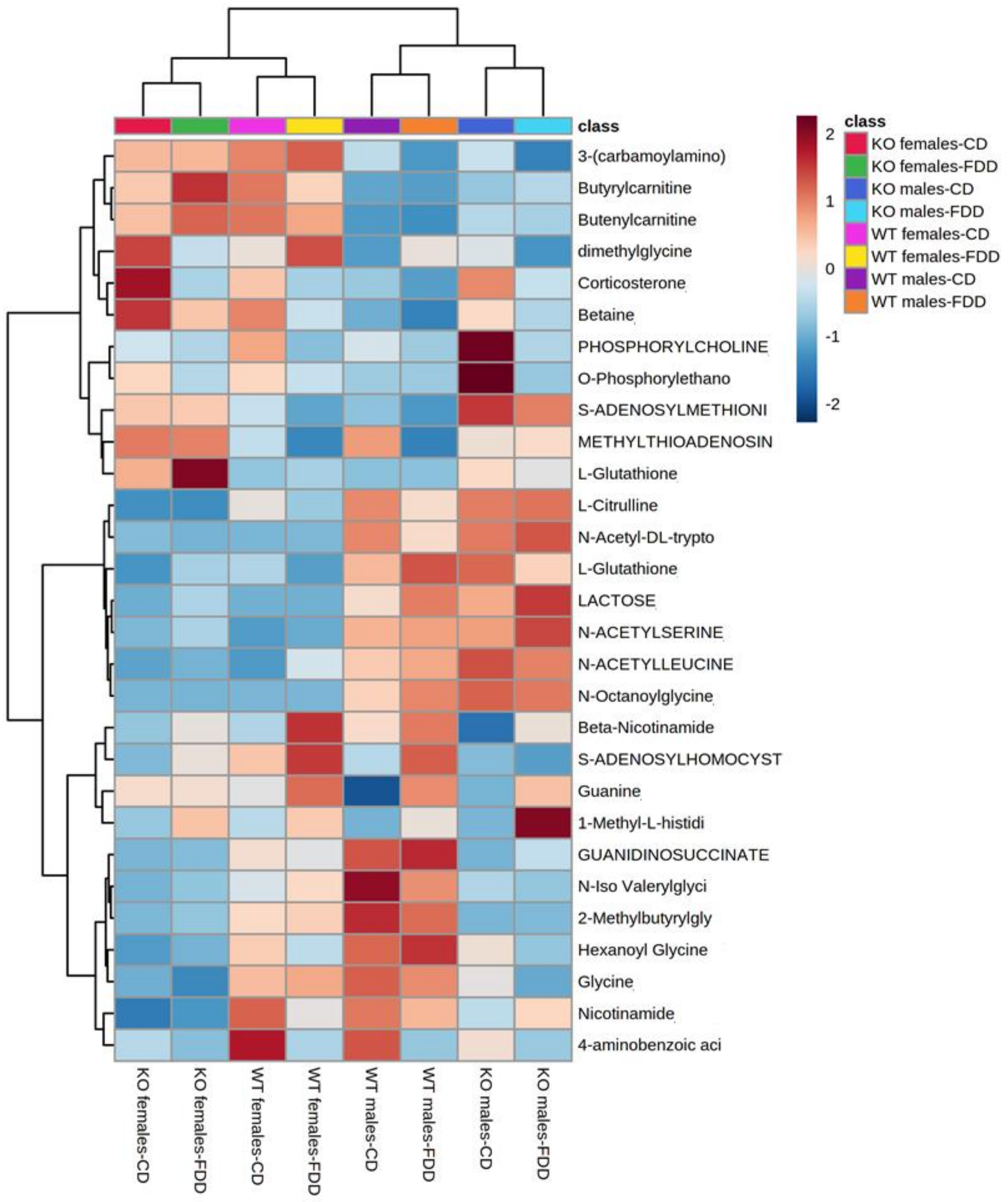

Supplementary Figure S7

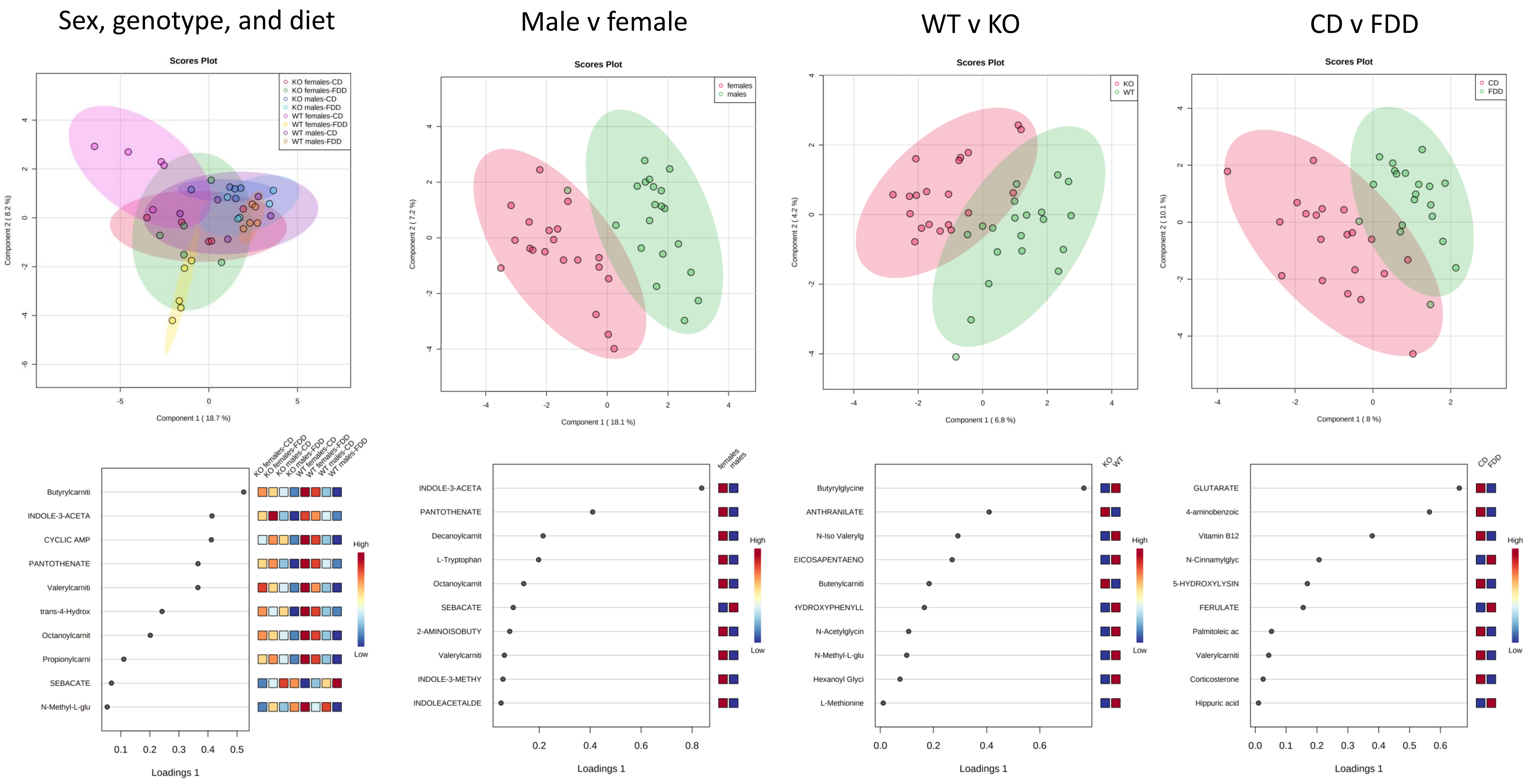

Supplementary Figure S8

All peaks

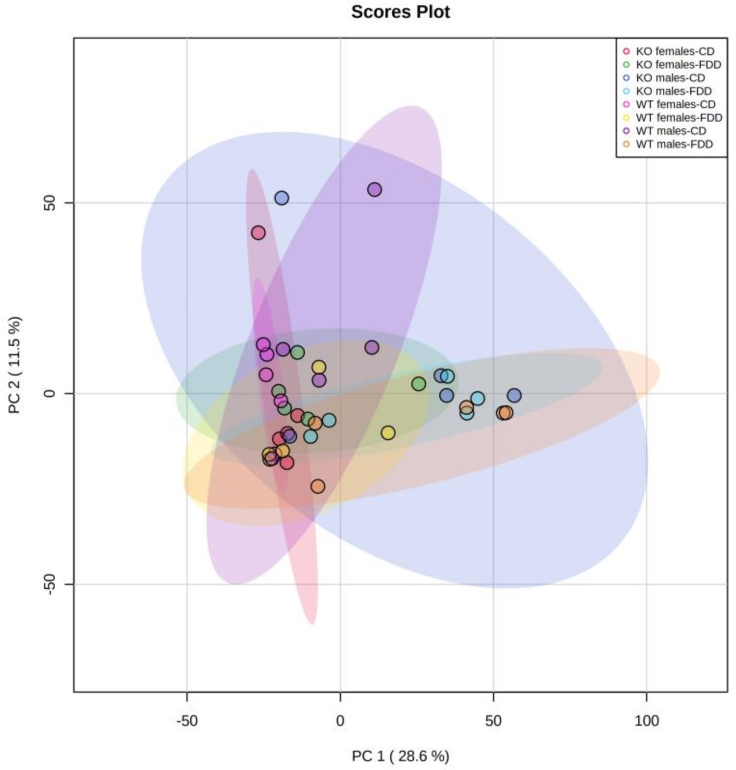

Identified Metabolites (OL1/OL2a)

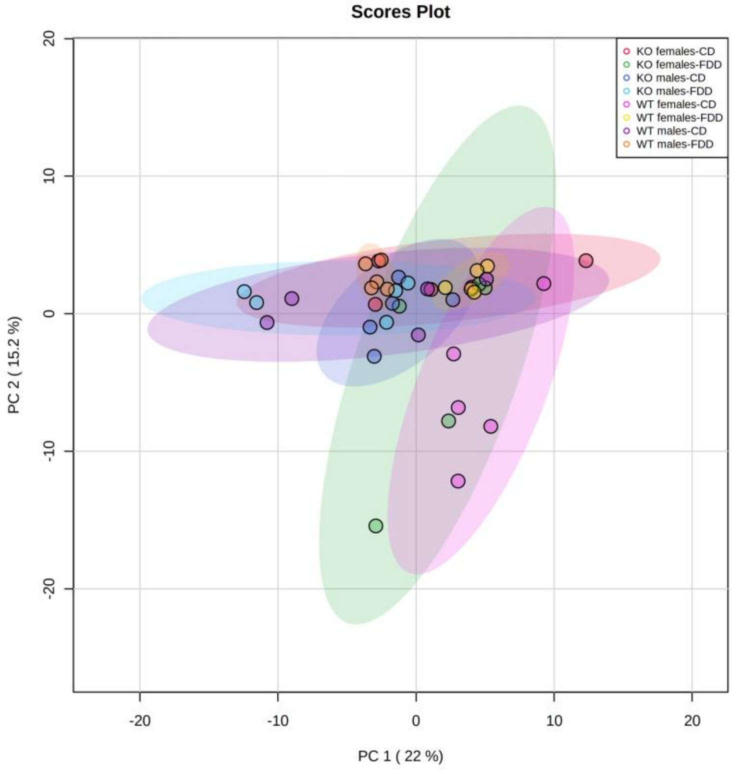

sPLS-DA selected metabolites

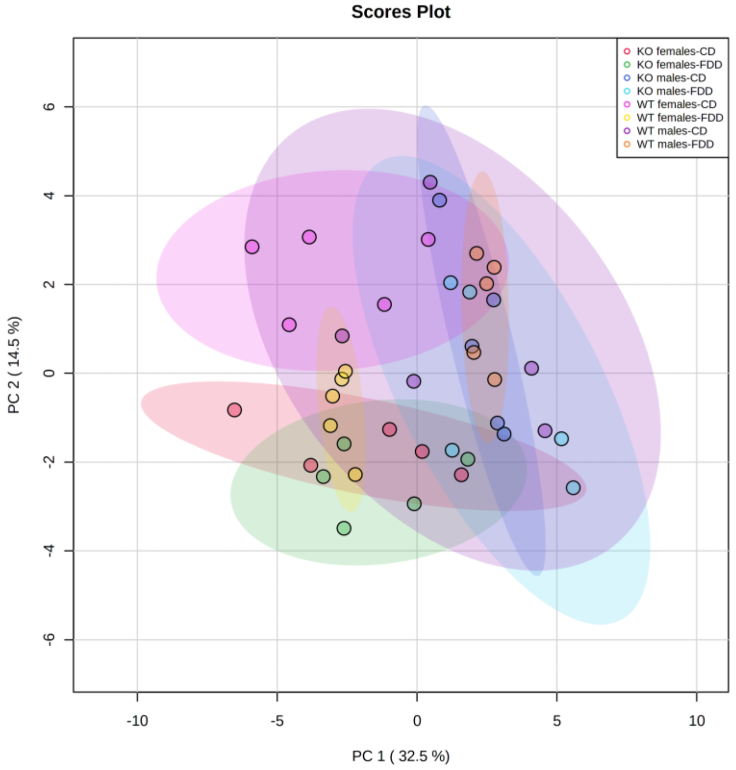

Supplementary Figure S9

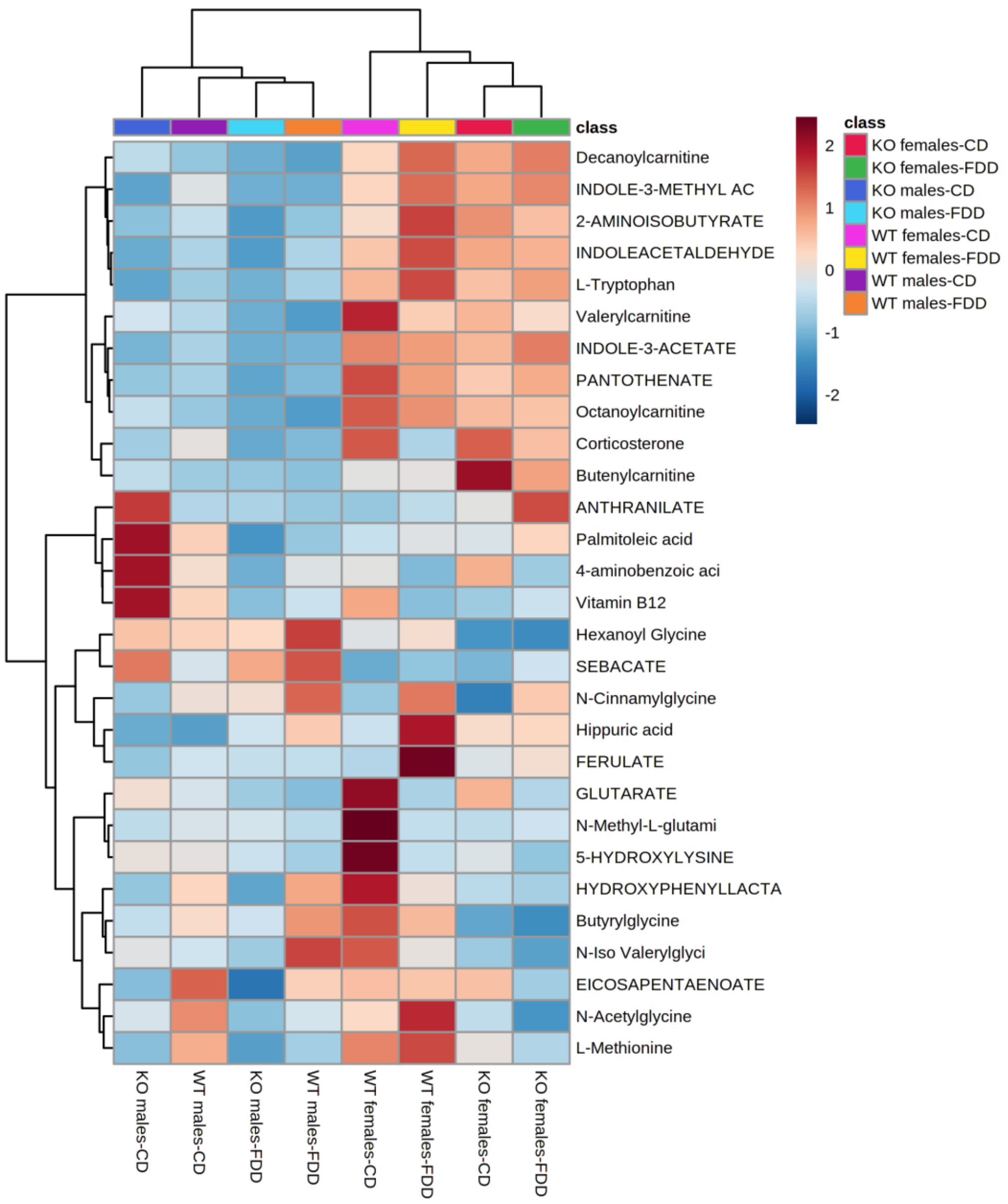

Supplement: Supplementary file 1 [file metabolites-12-00454-s001.zip › Supplementary file S2.pdf]
