# Supplementary figures and images for: Sex-Specific Metabolic Effects of Dietary Folate Withdrawal in Wild-Type and Aldh1l1 Knockout Mice
Source: Metabolites. 2022 May 18;12(5):454. doi: 10.3390/metabo12050454 (PMC9143804; doi:10.3390/metabo12050454)

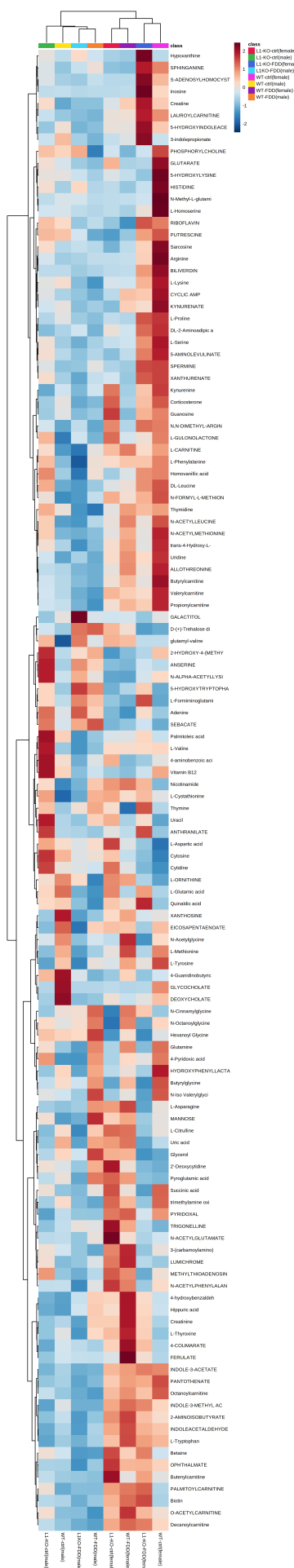

class

Supplement: Supplementary file 1 [file metabolites-12-00454-s001.zip › Supplementary file S4.pdf]
