# Supplementary material for: Sex-Specific Metabolic Effects of Dietary Folate Withdrawal in Wild-Type and Aldh1l1 Knockout Mice
Source: Metabolites. 2022 May 18;12(5):454. doi: 10.3390/metabo12050454 (PMC9143804; doi:10.3390/metabo12050454)
Supplement: Supplementary file 1 [file Supplementary file S3.pdf › Supplementary file S3.pdf]

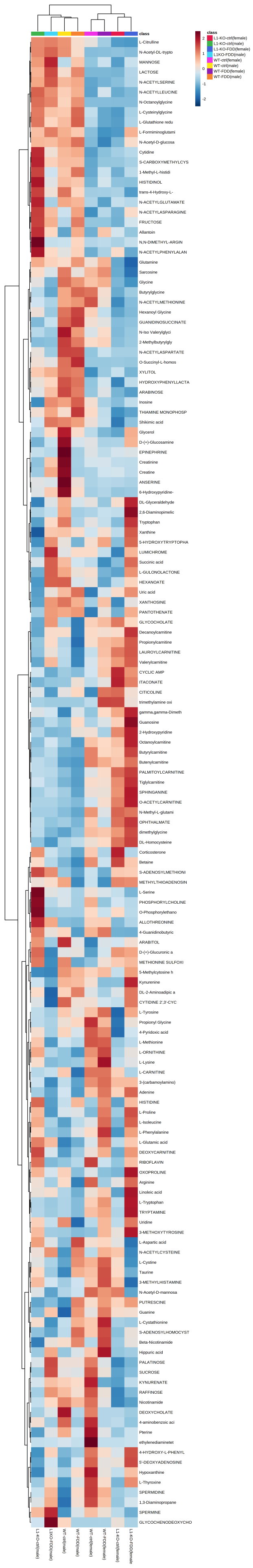
class
L1-KO-ctrl(female)
L1-KO-ctrl(male)
L1-KO-FDD(female)
L1KO-FDD(male)
WT-ctrl(female)
WT-ctrl(male)
WT-FDD(female)
WT-FDD(male)
2
1
0
-1
-2
L-Citrulline
N-Acetyl-DL-trypto
MANNOSE
LACTOSE
N-ACETYLSERINE
N-ACETYLLEUCINE
N-Octanoylglycine
L-Cysteinylglycine
L-Glutathione redu
L-Formiminoglutami
N-Acetyl-D-glucosa
Cytidine
S-CARBOXYMETHYLCYS
1-Methyl-L-histidi
HISTIDINOL
trans-4-Hydroxy-L-
N-ACETYLGLUTAMATE
N-ACETYLASPARAGINE
FRUCTOSE
Allantoin
N,N-DIMETHYL-ARGIN
N-ACETYLPHENYLALAN
Glutamine
Sarcosine
Glycine
Butyrylglycine
N-ACETYLMETHIONINE
Hexanoyl Glycine
GUANIDINOSUCCINATE
N-Iso Valerylglyci
2-Methylbutyrylgly
N-ACETYLASPARTATE
O-Succinyl-L-homos
XYLITOL
HYDROXYPHENYLLACTA
ARABINOSE
Inosine
THIAMINE MONOPHOSP
Shikimic acid
Glycerol
D-(+)-Glucosamine
EPINEPHRINE
Creatinine
Creatine
ANSERINE
6-Hydroxypyridine-
DL-Glyceraldehyde
2,6-Diaminopimelic
Tryptophan
Xanthine
5-HYDROXYTRYPTOPHA
LUMICHROME
Succinic acid
L-GULONOLACTONE
HEXANOATE
Uric acid
XANTHOSINE
PANTOTHENATE
GLYCOCHOLATE
Decanoylcarnitine
Propionylcarnitine
LAUROYLCARNITINE
Valerylcarnitine
CYCLIC AMP
ITACONATE
CITICOLINE
trimethylamine oxi
gamma,gamma-Dimeth
Guanosine
2-Hydroxypyridine
Octanoylcarnitine
Butyrylcarnitine
Butenylcarnitine
PALMITOYLCARNITINE
Tiglylcarnitine
SPHINGANINE
O-ACETYLCARNITINE
N-Methyl-L-glutami
OPHTHALMATE
dimethylglycine
DL-Homocysteine
Corticosterone
Betaine
S-ADENOSYLMETHIONI
METHYLTHIOADENOSIN
L-Serine
PHOSPHORYLCHOLINE
O-Phosphorylethano
ALLOTHREONINE
4-Guanidinobutyric
ARABITOL
D-(+)-Glucuronic a
METHIONINE SULFOXI
5-Methylcytosine h
Kynurenine
DL-2-Aminoadipic a
CYTIDINE 2',3'-CYC
L-Tyrosine
Propionyl Glycine
4-Pyridoxic acid
L-Methionine
L-ORNITHINE
L-Lysine
L-CARNITINE
3-(carbamoylamino)
Adenine
HISTIDINE
L-Proline
L-Isoleucine
L-Phenylalanine
L-Glutamic acid
DEOXYCARNITINE
RIBOFLAVIN
OXOPROLINE
Arginine
Linoleic acid
L-Tryptophan
TRYPTAMINE
Uridine
3-METHOXYTYROSINE
L-Aspartic acid
N-ACETYLCYSTEINE
L-Cystine
Taurine
3-METHYLHISTAMINE
N-Acetyl-D-mannosa
PUTRESCINE
Guanine
L-Cystathionine
S-ADENOSYLHOMOCYST
Beta-Nicotinamide
Hippuric acid
PALATINOSE
SUCROSE
KYNURENATE
RAFFINOSE
Nicotinamide
DEOXYCHOLATE
4-aminobenzoic aci
Pterine
ethylenediaminetet
4-HYDROXY-L-PHENYL
5'-DEOXYADENOSINE
Hypoxanthine
L-Thyroxine
SPERMIDINE
1,3-Diaminopropane
SPERMINE
GLYCOCHENODEOXYCHO
